# The Regenerative Applicability of Bioactive Glass and Beta-Tricalcium Phosphate in Bone Tissue Engineering: A Transformation Perspective

**DOI:** 10.3390/jfb10010016

**Published:** 2019-03-22

**Authors:** Baboucarr Lowe, Mark P. Ottensmeyer, Chun Xu, Yan He, Qingsong Ye, Maria J. Troulis

**Affiliations:** 1 School of Dentistry, The University of Queensland, Brisbane, Herston 4006, Queensland, Australia; babolowe@gmail.com (B.L.); chun.xu@uq.edu.au (C.X.); h.he@uq.edu.au (Y.H.); 2 Department of Radiology, Massachusetts General Hospital, Harvard Medical School, Boston, MA 02114, USA; mottensmeyer@mgh.harvard.edu; 3 Department of Oral and Maxillofacial Surgery, Massachusetts General Hospital and Harvard School of Dental Medicine, Boston, MA 02114, USA; mtroulis@mgh.harvard.edu

**Keywords:** Tricalcium Phosphate, Bioactive Glass, Bone, Scaffolds, Biomaterial

## Abstract

The conventional applicability of biomaterials in the field of bone tissue engineering takes into consideration several key parameters to achieve desired results for prospective translational use. Hence, several engineering strategies have been developed to model in the regenerative parameters of different forms of biomaterials, including bioactive glass and β-tricalcium phosphate. This review examines the different ways these two materials are transformed and assembled with other regenerative factors to improve their application for bone tissue engineering. We discuss the role of the engineering strategy used and the regenerative responses and mechanisms associated with them.

## 1. Introduction

The Tissue Engineering field has rapidly expanded over the past two decades with promising translational results [1]. Based on the growth in the numbers of publications, both the research and clinical foci in the field of bone tissue engineering (BTE) are enormous. This is particularly driven by current clinical limitations associated with the restoration of bone function such as immunological complications associated with allografts, donor site morbidity [2]. BTE approaches provide better solutions [3]. The most recent results in the area of stem bioprinting of biocompatible materials have many potential translational applications [4].

**Figure 1:**
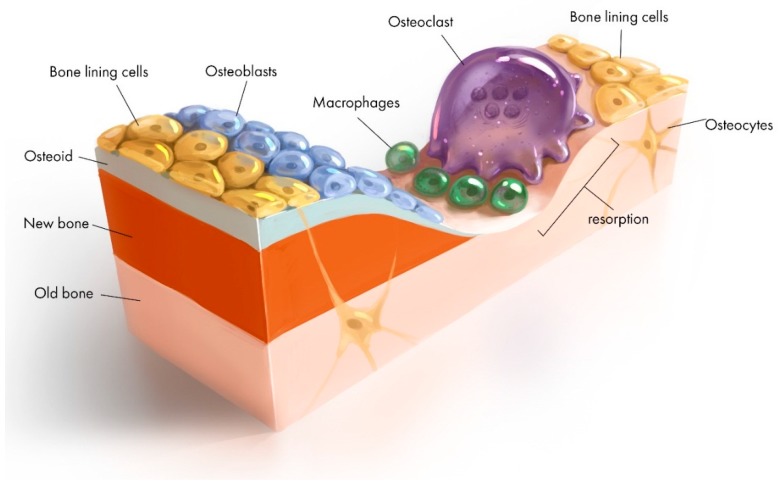
Schematic representation of bone remodeling showing bone lining cells (quiescent osteoblast) at first stage of activation; osteoclast (resportion); the resorptive lucanae where mononuclear cells differentiate into macrophage (reversal) and deposits of osteoid (formation) and osteocytes (maturation).

This paper focuses on two key biocompatible materials: Bioactive Glass 45S5 (BAG) and β-Tricalcium Phosphate (β-TCP) which have been used in BTE for decades [5,6,7]. Calcium Phosphate (Ca-P) materials have the potential to become the biomaterials of the future. They are safe, found naturally in our body, integrate with tissues easily and can be easily produced in large amounts at low cost [8]. It remains a great challenge to design and successfully apply biomaterials such that structural and functional restoration of native bone is accomplished, where by the remodelling of the biomaterial completely synchronizes with the natural healing process (as illustrated in Figure 1), and the degree of strength and stiffness required is maintained until new growing bone completely replaces and degrades [9]. Taken altogether, a discussion of the regenerative foci of synthetic grafts, highlighting the limitations and suggestion of approaches to improving their functionalities significance, deserves greater scientific attention. We discuss the strategic transformation of BAG and β-TCP the mechanisms associated with them, towards the development of different forms of scaffold matrices for applications in BTE.

Natural bone contains a hydrated calcium component, hydroxyapatite (HA) and therefore, if a material is able to form an HA layer in vivo may not be rejected by the body [5]. When Bioactive glass 45S5 is implanted, the glass reacts with native body fluid, forming apatite, after which it bonds to native bone and soft tissue, and releases calcium and silicon ions, which in turn enhance activation of osteogenic biomarkers and promote bone formation [10]. Chemically, sodium ions leach out of the glass, increasing local pH due to rapid exchange of sodium ions with H^+^ ions. This process initiates the dissolution of the SiO_2_ networks, later forming silanol groups, which gradually precipitates into a silica layer. The formation of this silica layer enhances the migration of the calcium and phosphate ions of the glass to form a dense layer of calcium phosphate. Further crystallization and integration of the hydroxyl and carbonate ions from the solution produces an apatite (hydroxycarbonated apatite) layer on the glass surface [11]. The collagenous fibers of the soft tissue can bond to this apatite and silica shortly after implantation [12]. There is evidence [13] of BAG attachment to soft tissue but the mechanism behind this process is unknown [14,15].

In calcium phosphates, it is believed that the release of the calcium and phosphate ions creates more precipitation of Ca-P, as a result of increasing ionic concentration. This facilitates protein adsorption and finally enhances bone formation [16]. Evidence of the role of proteins in the dissolution of Ca-P has been reported in Lui et.al [17], in which the dilution kinetics of Ca^2+^ ion species in Ca-P were much slower in bovine serum albumin coated surface compared to non-coated ones. A change in crystallinity was observed in protein-coated surfaces following reprecipitation. The role of proteins in the dissolution of Ca-P is dependent on three major factors: (1) properties of the bioceramic (phase, crystallinity, composition, and texture); (2) properties of the proteins (e.g conformation, isoelectric point, and composition) and (3) whether they act in solution or on a substrate. The proteins from the fluid of the local area mediate the ionic exchange process [18,19,20,21,22,23], and contribute to the dissolution events.

In vivo, the dissolution of calcium phosphate and reprecipatation are mediated by ions and proteins. The adsorbed protein can determine the final nature of the Ca-P crystal by either blocking or unblocking sites of nucleation of the substrate [24]. Given the roles of proteins in facilitating calcium nucleation [25], they have used as tactical entities in the construction of scaffold matrices in surface modified interfaces [26]. Despite the remarkable clinical results of bioceramic phosphates in BTE, the mechanisms associated with Ca-P induced bone formation are still obscure [8]. 

Nonetheless, the regenerative properties of BAG and β-TCP, as used in BTE, are governed by their chemical makeups and physical properties, which can strongly influence regenerative activators associated with repair and/or regeneration [27,28]. For some of the limitations associated with the applications of BAG and β-TCP in BTE, several other engineering strategies have been designed and applied to unleash their full regenerative capabilities. Among these limitations, 45S5 bioactive glass has slow degradation rates and this may complicate resorption and formation of new bone [29]. It is crystalline when sintered and this makes it challenging to apply in making porous scaffold matrices [30]. This limitation becomes particularly significant when controlling pore interconnectivity to construct and achieve a three dimensional geometric configurations [31,32]. 

In regards to β-TCP, rate of resorption does not correspond to the rate of bone formation. Although this is relative, the imbalance between resorption and osteogenesis have implications in the quality of new bone formed. Additionally, the brittle nature of β-TCP, lessens the toughness of constructs and their ability to withstand collapse [33]. The application of several other strategies, among those discussed below, are useful to alleviate some of these challenges.

Despite these limitations, the regenerative properties of β-TCP are quite distinct among other calcium phosphate and widely used in bone regeneration [34]. β-TCP is considered both osteoconductive and osteoinducive and its rate of resorption is higher compared to crystalline HA, which resorbs much slower [35,36]. Also, amorphous calcium phosphate has high rate of solubility and the rapid release of calcium and phosphates ions in aqueous can result in perturbation in the local pH, which negatively affects the adhesion and proliferation of cells as well their viability [35].

To apply both BAG and β-TCP in fabricating suitable scaffolds, the engineering strategies will involve: the transformation of the basic material properties (chemical or physical); designing the appropriate geometry; optimizing the ratio of the constituent polymer or co-polymer used to achieve mechanical stability; and constructing a unit three-dimensional porous architecture to facilitate tissue ingrowth and perfusion of blood. This is a particularly standard approach applied in the reconstruction of segmental defects [37]. Given, the strong connections between scaffold chemical and geometric nature and regenerative response, we focus our discussions on key areas of scaffold development and how transforming certain factors contribute towards robust scaffold properties and regenerative responses. Where applicable we discuss the translational indicators associated with the applications of these materials. Key subject areas in focus, related to the use of these materials, include porosity, bonding, surface area-to-volume ratio, mechanical properties, differentiation, and energy gradients based on material surface interaction. Additionally, other aspects in focus relate to surface modification, antibiotic delivery in multifunctional scaffolds, and translational models used to test the function of materials in bone repair or regeneration in BAG or β-TCP-based scaffold materials. 

## 2. Scaffold Transformation for Regenerative Application

To ensure that cells’ growth and regenerative capabilities are properly modulated and maintained, a scaffold should have one or more of these properties: osteoinduction (ability to induce stem cell differentiation to the bone lineage), osteoconduction (ability to maintain an optimum condition for neogenesis of the bone tissue), porosity, and mechanical stability [38,39,40,41]. From a performance-based perspective, some of these properties are more distinct than others in certain kinds of synthetic or natural materials. A selective transformation protocol allows us to enhance one or more properties to produce a support infrastructure for the cells to grow and foster regeneration. The ultimate focus in bone scaffold engineering is to be able to construct all the essential regenerative features in a single scaffold template that is eventually replaced with completely vascularized bone in a patient, irrespective of the bone defect size, shape and geometry. 

### 2.1. Scaffold Interface

A key area in the transformation of scaffold is the interface. This is where the “regenerative language” is first communicated and is a key determinant of the material’s regenerative behaviour. 

**Figure 2:**
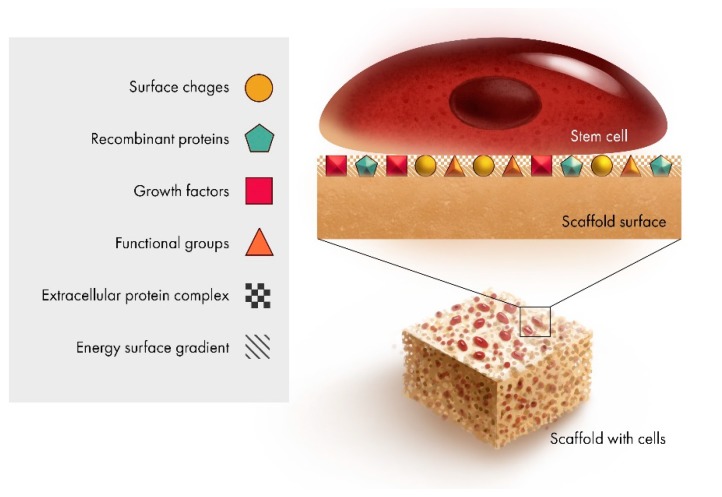
Schematic representation of the key regenerative factors at the scaffold interface.

It has been shown that the presence of a single protein (fibronectin) can greatly influence the surface charge factor and consequently the formation of apatite layers on the surface of BAG glass. It further shows that the presence of fibronectin produced electronegativity on the BAG surface and delayed the formation of both amorphous and crystalline apatite. The resultant increase in calcium ion concentration generated a structural change in the BAG surface. This shows that surface charge might play a significant role in protein-substrate interaction [42]. It has been found that the surface properties influence cellular adhesion and binding affinity of proteins to α5β1 integrin, in binding efficiency and strength of the interactions [43]. The regenerative implications are that the extra-cellular matrix (ECM) and α5β1 integrin signaling control the maintenance of bone formation capacity in stromal cells [44]. Key features at the interface as shown in Figure 2, include the surface area to volume ratio (topography), and the corresponding energy gradient produced. 

Other evidence of surface chemistry, gradient energy profiles, influence of pore size, topography, impact protein interactions and adhesion of bone cells have been reported [45]. Often, one or many of these properties are acquired or added to the material during the fabrication of the scaffold [46]. The modification of the scaffold interface is also aimed at achieving high-performance bonding/integration with the native tissue or cell when applied in vivo. Another aspect of the interface is aimed at improving the preferential affinity of the material surface to promote adhesion of the ECM. For example, biomaterials, which are cross-linkable due to their strong bonding affinity to functional groups, are good choices for surface modification. The regenerative implications of surface modification will be discussed in latter sections of this review.

Another important aspect of scaffold transformation takes into consideration the appropriate combination of toughness and maintenance of plasticity of the scaffold matrix in order to solve problems associated with behaviours of bioceramic particles, like β-TCP and BAG, when they are incorporated into polymeric interfaces. Given that the bioactive particles are initially covered with a polymer, they are not quickly exposed as the polymer degrades. Since cells can easily demonstrate preferential affinity to the bioactive particles, further degradation of the polymer may cause an inflammatory/localized body reaction as the scaffold degrades and the particles become exposed. Thus, sol-gel hybrid materials have been developed to soften bioactive glass for bone regeneration [47]. 

The regenerative sensitivity at the interface as a factor of regenerative response is not fully investigated. To do so will require development of high throughput protocols to understand changes in scaffold surface properties and how they impact regenerative susceptibility. This will provide more insight and predictability regarding the long-term regenerative profile of progenitor cells as applied in bone tissue engineering. Additionally, other key indicators of cell fate would be clearer.

### 2.2. Surface Modification

In the surface modification method, a monomer serves as the precursor for the desired functional group, serving as an initiator of the synthesis process. The concept of surface modification has been applied in several other engineering protocols for development of biomimetic scaffolds. The major surface modification techniques established and applied for the development of biomimetic scaffolds are: radiofrequency plasma deposition [48,49,50,51,52,53], silane modification of glass and ceramics [54,55] and thiol-based modification [56,57]. These forms of chemical interventions allow functional groups with regenerative effects (e.g., amine, carboxylic rings) to be successfully grafted to the underlying entity (e.g., another material forming part of the scaffold) to improve the regenerative potential of the scaffold [58]. Functional groups also influence the differentiation cascades of stem cells. Studies have demonstrated osteogenic differentiation of stem cells induced by the presence of amine functional groups [59,60,61]. To our knowledge, surface modification of β-TCP and BAG aimed at specifically assessing the role of a functional group via the insertion of functional rings for bone tissue regeneration has not been widely reported. However, Jiang et al., have demonstrated that surface modified BAG functionalized with amine for drug delivery also promotes formation of apatite layers and thus possesses regenerative potential for bone tissue engineering [62]. The osteogenic potential of amine-functionalized BAG is believed to be influenced by its positively charged surface and slow degradation rate [58]. Research into the area of functional group-based surface modification screening may lead to the development of highly mineralized scaffolds to maintain an ideal substrate-cell interaction profile and improve the conditions of differentiation and proliferation [63]. Also, a study by Keselowsky et al., showed that the amine functional group (–NH2), has more capability in adsorption of fibronectin compared to –COOH, and –OH groups [64]. 

The regenerative performance of surface modified materials is highly influenced by the hydrophilicity or hydrophobicity gradient established between the cell and the material. It is evident that cells have more preference to hydrophilic surface substrates than hydrophobic ones [65]. It is suggested that certain surface functional species of alkanethiols, carrying terminals such as –CH3, –NH2, –OH, or –COOH, affect both cell adhesion and protein adsorption [65,66]. Others have gone further to understand the activation process induced by these functional groups at the blood-material interface and revealed that surfaces with high contact activation have low adhesion of platelet rich plasma and blood. Hydrophobic-rich surfaces (–CH3, –COOH) showed no platelet adhesion. However, a significant shift was observed upon incubation with citrated plasma rich platelets, and adhesion was remarkably improved [67]. Others have shown that the hydrophobic gradient can be increased by the addition of BAG nanoparticles. The addition of BAG nanoparticles to a polycaprolactone film with an initial average contact angle of ~109.2° resulted in a significant decrease in contact angle, measuring a final value of 54.7° [68]. The contact angle can be measured using any of the OCA15 (Dataphysics), Optical Tensiometer (Dyne Technology, Lichfield, Staffordshire, UK), Contact Angle Meter (Holmarc Opto-Mechatronics Pvt. Ltd., Kochi, Kerala, India) or other machines. Given that β-TCP is a hydrophobic material, its water retention property can be improved by mixing with γ-PGA (poly (γ-glutamic acid)). Chemically, as the reaction proceeds, the α-C units off the carboxylic main chain form a hydrogen bond to the –OH groups of the β-TCP [69].

### 2.3. Cells 

**Figure 3:**
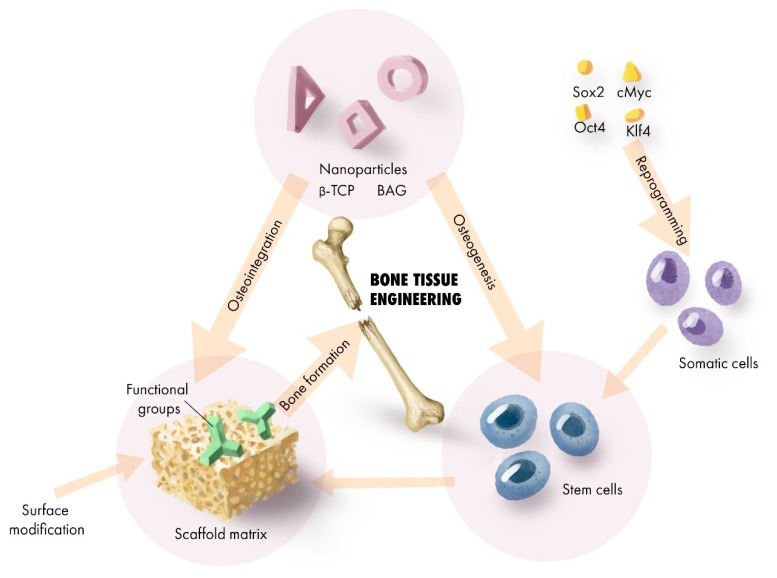
A summary of the transformation pattern associated with the regenerative applicability of β-TCP and BAG for bone tissue engineering.

Now that stem cells can be generated from somatic cells through reprogramming protocols based on signal transduction and key transcription factors [70]; adult somatic cells reprogrammed as induced pluripotent stem cells (iPSCs), as shown in Figure 3, can be another potential source of cells for bone tissue engineering application [71]. 

The applicability of this technology in tissue engineering, however, would require use of a suitable delivery mechanism of transcription factors to ensure minimal genomic alteration (e.g., insertional mutagenesis), largely associated with the use of delivery vectors [72]. A direct delivery method, of reprogramming proteins to produce induced pluripotency in somatic cells has been reported [73]. We suggest that by taking advantage of the controllable delivery properties of BAG and β-TCP and the ability turn them into microspheres or nanoparticles, a delivery method can be designed to deliver reprogramming proteins to minimize genomic alteration. Elsewhere, others have shown controllable delivery of protein unit by modification of the surface properties of bioactive glass [74,75].

### 2.4. Micro-Architecture 

Micro-architecture is a critical parameter of the scaffold and can significantly enhance regenerative performance. The scaffold’s microporous matrix, composed of interconnected, three-dimensional networks of pores, supports the adhesion and growth of cells. The microenvironment further supports the regenerative performance of the scaffold by promoting ingrowth of new bone and vasculogenesis. The porous micro-architecture of the scaffold is critical in supporting cell survival by meeting the mass-transport needs for nutrition, attachment, and migration [76]. Studies have shown that scaffolds with pore diameters greater than 300 µm enhance vascularization [77,78]. It is also maintained that scaffolds in which more than 60% of pores are between 150 µm and 400 µm and 20% them less than 20 µm, are ideal. Scaffolds with less than 1 µm pore size promote bioactivity and protein interaction. Porous materials with pore size ranging between 1 µm and 20 µm support cell growth, adhesion and migration. Moreover, pore diameters of 100 µm to 1000 µm enhance blood flow, bone ingrowth, and mechanical stability of the substrate [79]. Scaffolds with similar porous properties can be generated that are made of β-TCP-ion doped matrices [80], Polyacrolactone/β-TCP scaffold [81]. Other factors that influence porosity include material chemistry, fabrication, temperature, and chemical bonds formed between the constituent materials making up the scaffold. In this regard, particle size modelling and bonding chemistry, which are discussed further, have been creatively applied to improve scaffold regenerative properties and applicability in bone tissue engineering or regeneration. 

### 2.5. Nano-Architecture 

The fabrication of nano- size ceramic structures has been shown to improve bone regenerative performance. Nanoparticles have been used as target units to modulate the surface area to volume ratio and surface roughness of scaffolds [82]. β-TCP and BAG micro particles can be transformed to nanoscale length to improve scaffold regenerative performance and reinforce osteoinduction, mechanical stability, and the delivery profile of growth agents or antimicrobial agents as applied in bone tissue engineering. Cells co-cultured with BAG nanoparticles showed higher expression of prominent osteogenic markers ALP and RNX2, compared with other nanoparticles [83]. Additionally, for BAG, prepared using the sol-gel method, its nanoporosity significantly affected its rate of dissolution and silica release profile in simulated body fluid. These processes influence apatite nucleation and bioactivity [84]. The fabrication of PLLA nanocomposite using β-TCP nanoparticles as the building unit, demonstrated positive influence in porosity, compressive modulus, protein adsorption profile and affinity towards osteoblast adhesion [85].

### 2.6. Dopants 

Dopants facilitate the activation of important regulatory/molecular precursors (collagen I and osteocalcin) associated with early bone formation [86,87]. It is also reported that by introducing ion dopants, the structural mesoporosity of BAG, sheared by phosphorus pentoxide (P_2_O_5_), is changed. The mechanism regulating the change in structural geometry is due to the presence of P_2_O_5_, which propagates the formation of clusters of divalent cations and this impacts it regenerative property [88]. Others have doped TCP with metal oxides to improve mechanical strength, control resorption and osteoconduction. Results indicate stability and early stages of osteoid formation [89]. 

## 3. Application of Regenerative Scaffold for BTE and or Regeneration 

The many functional regenerative indicators of BAG and β-TCP are evident in how they are transformed. In a similar way, their applications are targeted to the direction at which they have been transformed. This will be discussed in the following sub sections.

### 3.1. Construct for Reconstruction of Segmental Defects 

**Figure 4:**
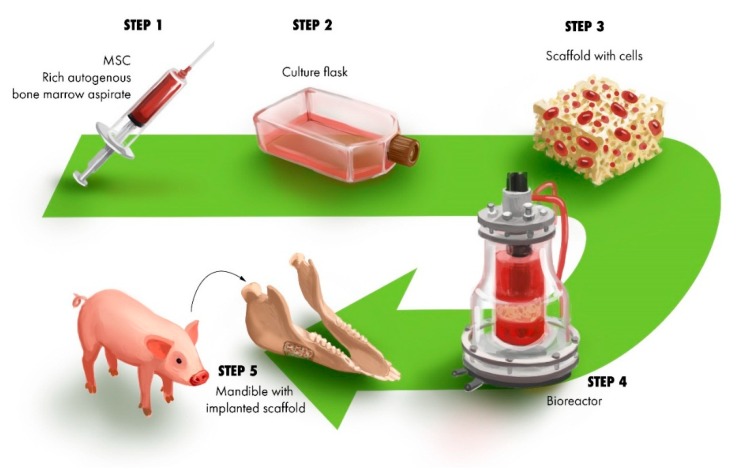
A schematic summary of the process of mandibular reconstruction in minipig model. An autogenous aspirate of bone marrow cells is harvested (**1**) and expanded (**2**). Cells are seeded to scaffold (construct) (**3**); Incubation in bioreactor (**4**); Construct is implanted into a critical size defect of the pig mandible (**5**).

A reconstructive strategy has been presented for the repair of canine mandibular bone defects with stromal cells delivered on a mechanically stable scaffold made of β-Tricalcium Phosphate. The stromal cells, osteogenically treated in dexamethasone, β-phosphoglycerol media prior to interacting with scaffolds, maintained their osteogenic phenotype [90]. The resorbability of the substrate prevents the implant from remaining in the bone tissue. This study have demonstrated that incorporating rod shaped β-TCP particles produced evidence of improved bone development [91]. However, it is worth noting that rapid resorption of the substrate may inhibit proper bone tissue deposition and thus an appropriate balance between bone formation and scaffold resorption needs to be maintained. It has been speculated that proper bioresorption depends on the biological activity of the recipient bone cells and as such may vary between different cells types [91]. An approach to reconstruction of a critical size mandibular defect in minipig model is shown in Figure 4. Early implantation was made of scaffolds seeded with autogenous cells, treated with osteogenic factors and incubated in a rotational oxygen-permeable bioreactor for 2 weeks prior to implantation.

Results showed that cell penetration decreased as extracellular matrix deposition progressed. Although animals were sacrificed at a relatively short time point, angiogenesis was observed in the centre of the construct and was more prominent in areas of newly formed bone. This study also confirmed that early implantation of constructs was beneficial [81]. The propagation of angiogenesis in the center of constructs is still a critical challenge to achieve complete regenerative reconstruction of large segmental defects. To solve this challenge, and promote neovasculation at the center, others have introduce a method to activate the hypoxia inducible factor (the master regulator of angiogenic and metabolic response to low oxygen) using deferoxamine [92]. Others studies have shown that the activation of this pathway accelerates bone regeneration [93]. The injection of angiopoietin 2 into the defect area has also shown evidence of promoting bone repair by accelerating angiogenesis in synthetic grafts This concept is based on the function of the angiopoietin tyrosine kinase receptor 2 pathway in tumor cell survival and metastasis, which is characterized by active angiogenesis [94]. In another study, 3D printed polybutylene terephthalate scaffolds coated with micro-size β-TCP were infiltrated with endogenous mesenchymal stem cell and grown in a bioreactor and successfully facilitated the bridging of femoral critical size defects (42 mm). This phenomenon was not observed in grafts free of mesenchymal stem cell and β-TCP, which validates their role in regeneration [95]. Elsewhere, others have shown the application of N-hydroxysuccinimide as an adhesive material [96]. By binding free bioglass particles a more organized matrix for reconstruction of critical size defects can be constructed. As shown in this study, chondroitin sulfate was effectively combined with 45S5 bioglass and bone marrow cells for repair of distal femoral bone defect with promising results [96]. This technique has the potential to reduce inflammation emanating from free particulate migration from constructs, in vivo and stabilize bioceramic-based scaffold matrices.

### 3.2. Delivery

BAG nanoparticles can serve as a reservoir for biological molecules, and they and their nanointerface can facilitate adhesion of osteoprogenitor cells and promotion of vascularized bone ingrowth. The physical properties of the glass can be optimized to target other biologic responses associated with bone development [97]. BAG’s functional capabilities are not limited to bone growth function, as BAG can control microbial activity around the defect and this promote proper healing [98]. A study on S53P4 bioactive glass, demonstrated bactericidal effects on several clinically important panels of anaerobic bacteria. The mechanism of the bactericidal effect was due to the release of the cations in the aqueous medium. When the cations are released, pH and osmotic pressure increase and partly explain the growth inhibition effect. The high concentration of alkali and calcium ions may also have been responsible for the perturbation of the membrane potential of bacteria [30,99].

Comparatively, β-TCP does not elicit a bactericidal effect in its natural form. However, β-TCP can be further processed for the efficient load and delivery of antibiotics. The fabrication of microporous structural units of β-TCP can provide stability to deliver antibiotic compounds. The maintenance of stability during loading and delivery was more prominent in β-TCP microporous constructs compared to powder or granular forms of β-TCP, which are too unstable for delivery purpose [100]. The functionalization of β-TCP with oxides of zinc and silver can achieve dual functionality, serving as a regenerative substrate and antimicrobial substance [101,102].

Additionally, by modifying the structural geometry of the material, drug molecules can be systematically incorporated and released using β-TCP or BAG. This approach has quite useful prospective translational benefits as a drug delivery system. It has the benefits of sparing patients from the adverse effects of using non-degradable PMMA (poly (methyl methacrylate)) beads, for which a second operation is required to remove them after the antibiotics have been released [100]. Additionally, given that the risk of impairment of bone regeneration due to bacterial colonization is high and can cause necrosis of tissue (especially periodontal), the role of β-TCP and BAG applied as resorbable delivery agents is an important one.

The articles referenced in Table 1, outlined different methods of transforming β-TCP to serve as an antimicrobial delivery agent. It should however, be noted that the excellent stability and delivery response profiles demonstrated by these materials (BAG and β-TCP) were not exclusively tested or investigated for regenerative applicability in bone tissue damage repair and/or regeneration. 

However, from a material science perspective, authors provided vital knowledge about the technical processing of β-TCP, which could be applied towards the engineering of scaffolds for BTE. 

### 3.3. Reinforcement of Stability in Guided Bone Regeneration (GBR) Membrane

Both BAG and β-TCP also been selectively applied in GBR. Exhaustive analyses and review of GBR are captured by [105,106,107]. The concept of guided bone regeneration was first introduced by Hurley et al. in 1959 [108]. In GBR, a membrane is applied around the defect area, which assists in containing blood clot and separating the defect site from the bordering connective tissues, thereby providing space for formation of new bone cells and regeneration of the defect [109].

Currently, both synthetic and naturally derived resorbable membranes for GBR are commercially available. The synthetic materials include PGA (polyglycolic acid), PLA (polylactic acid), PCL (polycaprolactone) and their derivatives or copolymers. These membranes are clinically beneficial owing to their excellent biocompatibility, biodegradability and tissue integration properties. However, such polyester-based membranes may lose mechanical strength when incubated during cell culture. 

To solve this drawback, BAG or β-TCP have been used to reinforce membrane stability and improve membrane bioactivity for GBR. Other naturally derived membranes, such as collagen, have poor mechanical stability, and uncontrollable degradation rates. Their derivation from mainly animal or human origin increases the risk of disease transmission [106,107,110]. As a result, the development of other resorbable, biocompatible membranes of natural origin, like chitosan and alginate, have gained attention [105]. In Table 2, recent studies investigating β-TCP for guided bone regeneration in animal models have been summarized.

In another study, the increase in tensile strength in a PLLA membrane resulted from the addition of strontium borosilicate bioactive glasses. This response was created as cations derived from the microparticles cross-linked with the carboxyl groups of the PLLA. The bonding triggers an interlinkage in the membrane and yields a corresponding increase in Young’s modulus [117]. 

The combination of β-TCP with recombinant human growth factor, applied to improve the performance of a Bio-OSS™ (Geistlich Pharma, Wolhusen, Switzerland ) membrane, was aimed at increasing bone mineral density. The growth factors profoundly mediated the regulatory signal associated with growth and differentiation of the cells. The addition of β-TCP fostered both resorption of the membrane and quality of new bone [116]. In a different study, β-TCP blended with PLGA (poly (lactic-co-glycolic acid)) and PCL displayed increased elastic modulus and surface roughness properties in a 3D printed membrane. The membrane was applied over buccal defects, aimed at preserving the underlying tissue, and protecting the area from exposure to blood, saliva and irrigation [112].

### 3.4. Reinforcement of Mechanical Stability

The mechanical stability can be enhanced by incorporation of ceramic particles likes BAG. A study by Li et al. (2015) shows increased surface roughness, hydrophilicity, and flexibility in chitosan-based GBR membrane, after incorporation of BAG and PHBV (poly (3-hydroxybutyrate-co-3-hydroxyvalerate)) microspheres. The membrane promoted cell adhesion, ALP activity, sustained and controlled release of drug molecules. The applicability of these kinds of membrane may be useful in GBR of periodontitis where mechanical stability to prevent failure is essential. In a similar vein, given that BAG can be applied in regeneration of hard or soft tissue, it can be a remarkable candidate for periodontal application [12,118]. 

Towards the development of a gelatin- β-TCP scaffold, the β-TCP mainly contributed to the mechanical strength of the membrane. This was prepared by initially making a slurry of gelatin-β-TCP, which was later lyophilized and cross-linked. The membrane was more than twice as strong as a collagen membrane and its ALP activity also increased. This biological response was closely associated with hydrophilicity, and reinforced osteoconductivity of the membrane due to the β-TCP. Histology results confirmed bone formation. However, there was no significant difference in bone volume between the two membranes when compared to the untreated control [115]. 

## 4. Summary and Conclusion

One key challenge remains, which is specifically how to institute all the essential regenerative features in a single scaffold template that is converted to the patient’s own vascularized bone. To the extent that we defined the different parameters of constructing or reconstituting a bone-regenerative scaffold by different methods, it is worth noting that these parameters are not independent of each other, especially during the fabrication process of the scaffold. The aim is to give a technical perspective on how to interlock and combine a smart engineering strategy to assure stability, regenerative sensitivity and enrichment of substrates. 

Therefore, exploiting these forms of design is expected to produce a robust method for designing scaffolds especially in co-construct development where one modelling strategy might not be sufficient for creating a substrate infrastructure with multifunctional capabilities. The regenerative applicability of bioactive glass and β-tricalcium phosphate largely stems from their physiological reactivity, cyto-sensitivity and influences on the cascades of stem cell differentiation and development of new bone tissue. Both β-TCP and BAG are easily transformed into different forms and shapes, embedded in polymer matrices, and sandwiched with other growth factors to improve their application potentials in the field of bone tissue engineering. 

To design a sacrificial template for the restoration of bone defects, these materials serve as regenerative entities that modulate the regenerative capabilities of biomaterial-based substrates in a variety of ways. The research covered in this article includes both in vitro and in vivo studies. The assessments provide a strategic outlook in the role of these materials and how they are strategically used to achieve bone regeneration. We discussed the use of these materials and their roles in addressing drawbacks and reinforcing the capabilities of other polymers, applied in bone tissue engineering. We focused our discussions on parameters including porosity, surface area-to-volume ratio, mechanical stability, initiators of differentiation cascades, charge gradients based on material surface-cell interaction, reinforcement of stability (both mechanical and regenerative) of substrates, functional-group-based surface modification, antibiotic delivery in multifunctional application of scaffolds, translational applicability in different screening models (in vivo) as well as a justification of the engineering strategy used, where applicable and necessary for better understanding of the concept-based design. 

Finally, for biomaterials of the future, it will be compelling to apply advanced tools of molecular biology, e.g., genomic sequencing, and proteomics to understand the ‘regenerative language’ of stem cells when combined with these materials and how the transformation of their functional properties affects the sequence of biological events associated with repair or regeneration. Additionally, the design of in vitro and in vivo models to further investigate the resorptive properties of β-TCP and bioactive glass-based scaffolds, will offer more clues into the complex mechanism of biomaterial resorption, in regenerative reconstruction of large defects. This is a still a major challenge in the field.

## Figures and Tables

**Table 1:** The processing and application of β-TCP with other materials as agent for bacterial control.

Material	Method	Form	Activity Test	Observations	Ref.
Ag/β-TCP	Doping	Nanoparticles Pore interconnectivity with even distribution of macropores.	L929 cells; S. epidermidis and S. aureus	Significant inhibition of bacteria and no toxic effect to fibroblast cells	[101]
PEG/β-TCP	Plasma polymerization	Disc	S. aureus	Exhibited a controlled release profile of antimicrobial drug and showed astrong bactericidal effect	[103]
Zn/β-TCP	Sol-gel	Nanoparticles Nanoparticle size (10-500nm)	S.aureus, E. coli, S. typhi	Zn-β-TCP (3.2wt% of Zn) showed the most antibacterial activity	[104]

^1^ Zn (zinc); PEG (polyethylene glycol); Ag (silver); PLA (polylactic acid).

**Table 2:** In vivo studies of composites comprising β-TCP for guided bone regeneration.

Material	Fabrication	Model/Defect	Time Points	Results	Ref.
rhBMP-2/PCL/PLGA/β-TCP	3D printing	Calvaria, rabbit	8 weeks	Bone turn over significantly higher than control group (*p* < 0.05). Bone to implant contact ratio significantly higher (*p* < 0.05). Full or partial absorption of implant observed.	[111]
PCL/PLGA/β-TCP/rhBMP-2	3D printing	Lower Jaw, Beagle Dog	4 to 8 weeks	The stability of the membrane was maintained at 4 weeks (post-implantation). Complete healing; new bone deposition observed. Significant increase in bone formation from 4 to 8 weeks. No inflammatory reaction.	[109]
PCL/PLGA/β-TCP	3D printing	Extracted premolars; mandibular alveolar ridge; Beagle Dog	8 weeks	Higher levels of new bone area and bone implant contact, compared to the control (collagen membrane). Remaining biomaterial was much higher compared to control. Results were insignificant to each other.	[112]
Modified Silk/β-TCP	Casting, particle deposition	Rabbit; Calvaria	5 and 10 weeks	Control (collagen membrane) Rate of resorption higher in control compared to membrane. Silk/β-TCP highly support bone formation and restoration of microarchitecture of defect compared to the control. Less statistical difference from histomorphometric data between the two (*p* < 0.05).	[113]
β-TCP/HA granules	N/A	Minipig; Lower premolar	3 and 8 weeks	Group with higher percentage of β-TCP (90%) showed more mineralized bone.	[114]
Gelatin/β-TCP	Freeze-dried/Cross-linking	Calvaria, Rat	2, 4, 8 weeks	Bone volume was higher in Gelatin/β-TCP membrane compared to control. Absorption was greater in collagen compared to Gelatin/β-TCP. No significant difference in bone volume between collagen membrane and Gelatin/β-TCP.	[115]
Bio-Oss/β-TCP/rhPDGF	N/A	Calvaria; Rat	2, 4, 6, 8, 10 weeks	Significant increase in bone mineral density. A 30% reduction in mean volume of remnant bone particles.	[116]

^2^ PCL (polycaprolactone); rhPDGF (recombinant human platelet-derived growth factor); N/A (no explicit description of the material development process).
